# Salidroside protects against ventilation-induced lung injury by inhibiting the expression of matrix metalloproteinase-9

**DOI:** 10.1080/13880209.2021.1967409

**Published:** 2021-09-13

**Authors:** Hui Zhang, Wenwen Dong, Siyuan Li, Yunqian Zhang, Zhou Lv, Lu Yang, Lai Jiang, Tao Wu, Yan Wang

**Affiliations:** a Department of Anesthesiology and Surgical Intensive Care Unit, Xinhua Hospital, Shanghai Jiaotong University School of Medicine, Shanghai, China; b The Key Laboratory of Exercise and Health Sciences of Ministry of Education, School of Kinesiology, Shanghai University of Sport, Shanghai, China; c School of Medicine, Shanghai Jiaotong University, Shanghai, China

**Keywords:** Acute lung injury, endothelial dysfunction, NF-κB signalling pathway

## Abstract

**Context:**

Salidroside, a compound extracted from *Rhodiola rosea* L. (Crassulaceae), possesses many beneficial pathological effects.

**Objective:**

To explore the effect of salidroside on ventilator-induced lung endothelial dysfunction *in vivo* and *in vitro*.

**Materials and methods:**

*In vivo*, male ICR mice were divided into sham, ventilation, salidroside, and ventilation plus salidroside groups. The mice were ventilated for 4 h, salidroside (50 mg/kg) was administrated intraperitoneally before ventilation, dexamethasone (Dex) (5 mg/kg) was used as a positive control. *In vitro*, mouse lung vascular endothelial cells (MLVECs) were treated with salidroside, MMP-9 siRNA, and BAY11-7082 (10 μM), and then exposed to cyclic stretch for 4 h. Afterward, lung tissues and MLVECs were collected for further analysis.

**Results:**

Salidroside pre-treatment significantly reversed the expression of vascular endothelial cadherin (VE-cadherin) and zonula occluden-1 (ZO-1) proteins in cyclic stretch-treated MLVECs (0.46 ± 0.09 *vs.* 0.80 ± 0.14, 0.49 ± 0.05 *vs.* 0.88 ± 0.08) and ventilated lung tissues (0.56 ± 0.06 *vs.* 0.83 ± 0.46, 0.49 ± 0.08 *vs.* 0.80 ± 0.12). The results further indicated that salidroside inhibited the expression of matrix metalloproteinase-9 (MMP-9), whereas knockdown of its expression restored the expression levels of VE-cadherin (0.37 ± 0.08 *vs.* 0.85 ± 0.74) and ZO-1 (0.48 ± 0.08 *vs.* 0.81 ± 0.11) in stretched MLVECs. Meanwhile, salidroside inhibited the NF-κB signalling pathway and alleviated lung injury.

**Conclusions:**

Salidroside protected against stretch-induced endothelial barrier function, improving lung injury after ventilation. Thus, salidroside may be a promising therapeutic agent for patients with MV-induced lung injury.

## Introduction

Mechanical ventilation (MV) is a life support therapy for patients suffering from respiratory diseases. Despite its life-saving potential, it may also induce or exacerbate lung injury, which leads to ventilator-induced lung injury (VILI) (Goligher et al. 2015). VILI results from constant cyclic stretching and is accompanied by the induction of inflammatory response. This condition also involves endothelial cell damage and increased alveolar-capillary permeability (Kneyber et al. 2014). Although previous evidence has shown that low tidal volume ventilation can improve the clinical outcome of these patients (Tobin 2000), a treatment strategy for VILI has not been developed, to the best of our knowledge.

Interendothelial junctions are composed of protein complexes of adherens junctions (AJs), tight junctions (TJs), and gap junctions. Among them, zonula occluden-1 (ZO-1) and vascular endothelial cadherin (VE-cadherin) are two significant interendothelial junction proteins. Under pathophysiological conditions, the integrity of the endothelium can be disturbed, resulting in the disruption of junction proteins. Matrix metalloproteinases (MMPs) are members of the family of calcium-dependent zinc-containing endopeptidases. These enzymes can cleave the majority of matrix proteins, as well as several non-matrix targets, including cytokines, chemokines, adhesion molecules, and surface receptors (Stamenkovic 2003). It is interesting to note that previous studies have found that the expression levels of certain MMPs, notably MMP-9, a member of the gelatinase subfamily, are elevated in the plasma of patients with acute lung injury (ALI) and acute respiratory distress syndrome (ARDS) (Hsu et al. 2015). Upregulated expression levels of MMP-9 have also been reported to be involved in several experimental lung injury models (Renckens et al. 2006; Lukkarinen et al. 2009; Wang et al. 2012). However, the role of MMP-9 in different lung injury models, notably in VILI, remains controversial. It has been suggested that inhibition of MMP-9 activity could be beneficial in models of sepsis-induced ARDS (Steinberg et al. 2005) and VILI (Foda et al. 2001; Kim et al. 2006) by decreasing neutrophil-mediated inflammation. In contrast to these findings, studies with mice lacking MMP-9 have shown aggravated pulmonary injury induced by sepsis (Renckens et al. 2006) and MV, indicating that MMP-9 exerts a protective effect in ALI (Albaiceta et al. 2008).

Salidroside is one of the effective ingredients of *Rhodiola rosea* L. (Crassulaceae), which has been widely used as a traditional herb in Asia and Eastern Europe (Mattioli et al. 2009). Accumulating evidence has shown that salidroside possesses several pharmacological properties, such as anti-inflammatory (Wang et al. 2015) and antioxidant activities (Zhang et al. 2007), as well as cardioprotective (Zhu et al. 2015) and neuroprotective effects (Wang et al. 2015). It has also been reported that salidroside alleviates LPS-induced lung injury (Guan et al. 2012) and caecal ligation and puncture (CLP)-induced sepsis in mouse models (Lan et al. 2017). However, the effects of salidroside on endothelial permeability remain unknown. As a consequence, the present study investigates the pharmacological role of salidroside in mechanical stretch-induced endothelial dysfunction and ventilation-induced lung injury.

## Materials and methods

### Microarray data collection

The present study used the Gene Expression Omnibus (GEO) database (http://www.ncbi.nlm.nih.gov/geo/) to retrieve expression profile datasets. The search term used was: ‘ventilation lung’.

### Animal experiments

Healthy, pathogen-free male mice were obtained from the Institute of Cancer Research (ICR). Male ICR Mice (weight, 25–30 g) were housed in an environmentally controlled room (temperature, 24 ± 2 °C; humidity, 55 ± 10%) with a 12 h light/dark cycle and free access to food and water. All experiments were performed according to the guidelines for the care and use of animals as established by the Animal Ethics Committee of Xinhua Hospital, Shanghai Jiaotong University School of Medicine (approval no. XHEC-NSFC-2020-038). Following anaesthesia with ketamine (70 mg/kg) and xylazine (10 mg/kg) by intraperitoneal administration (Xu et al. 2007), the animals underwent tracheotomy. A 20-gauge blunted needle was inserted into the trachea and the mice were ventilated under volume mode (30 mL/kg; 70 breaths/min; inspiratory expiratory ratio: 1:2) for 4 h using a small animal ventilator (Inspira; Harvard Apparatus Ltd.). Totally 64 male mice were randomly divided into the following four groups: (i) Sham; (ii) ventilation group; (iii) salidroside; and (iv) ventilation and salidroside. The Sham group only underwent tracheotomy. Salidroside (50 mg/kg; Sigma–Aldrich; Merck KGaA) was injected intraperitoneally according to the former study published by our group (Wang et al. 2017). Dex (5 mg/kg, Cisen Pharmaceutical Co., Ltd. Jining, China) was used as a positive control. Following ventilation, all animals were sacrificed instantly and 1 mL of blood was collected from the heart. The mice weighed between 25 and 30 g at the time of sacrifice, and one series of mice were sacrificed for BAL Fluid assay and another parallel series was collected for histological and further analysis.

### Histological examination

The lung specimens were fixed with 10% formalin, embedded in paraffin, and cut into 4 μm sections. The sections were stained with haematoxylin and eosin and examined with light microscopy. The degree of lung injury was analyzed by two pathologists blinded to the study, based on the degree of inflammation and thickness in the alveolar wall using the following scoring system (Zhang et al. 2015): 0, normal tissue; 1, small inflammatory changes; 2, mild to moderate inflammatory changes; 3, moderate inflammatory injury; 4, moderate to severe inflammatory injury; and 5, severe inflammatory injury.

### Immunofluorescence analysis

Lung paraffin sections (5 µm) were dewaxed and placed in a microwave box with a certain pH 6.0 citrate buffer for the antigen retrieval. After incubation with 5% BSA at room temperature for 30 min, lung sections were incubated with primary antibodies against MMP-9 (cat.no. ab38898; 1:200; Abcam), ZO-1 (cat.no. 21773-1-AP; 1:200; ProteinTech Group, Inc.), VE-cadherin (cat.no. ab205336; 1:200; Abcam), CD31 (cat.no. GB11063-2, 1:200, Servicebio Technology, Wuhan, China) overnight. Secondary antibodies corresponding to each primary antibody (CY3: goat anti-rabbit, cat.no. GB21303, 1:300; Servicebio; Alexa Fluor 488: goat anti-mouse, cat.no. GB25301, 1:400; Servicebio) were incubated in dark for 1 h and counterstained with 4′,6-diamidino-2-phenylindole (DAPI) (cat.no. C1005, Beyotime Institute of Biotechnology, Shanghai, China). Images were obtained by using a fluorescence microscope (Olympus BX53, Japan).

### Lung wet-to-dry weight ratio

Following ventilation for 4 h, the right upper lungs of the mice were weighed and dried at 60 °C for 48 h. The ratio of wet/dry weight (W/D) was calculated.

### Preparation of bronchoalveolar lavage fluid (BALF)

BALF was collected from the lung by infusion with 1 mL ice-cold PBS thrice. BALF samples were centrifuged at 1500 *g* at 4 °C for 10 min. Following centrifugation, the cell pellets were resuspended with PBS for cell counting and the BALF supernatant was used for detection of the total protein concentration.

### RNA interference

MMP-9 small interfering RNA (siRNA) was synthesized by Genepharm Biotech Corp. The sequences of the siRNAs were as follows: MMP-9 siRNA sense, 5′-GCCAGACACUAAAGGCCAUTT-3′ and antisense, 5′-AUGGCCUUUAGUGUCUGGCTT-3′. Control siRNA consisted of a scrambled sequence without any specific target. Transfection of siRNA was performed using the Xfect siRNA transfection reagent (Takara Bio, Inc.).

### Cell culture and treatment

Mouse lung vascular endothelial cells (MLVECs) were isolated from 48 male mice (2–4 weeks) as previously described (Dong et al. 2015). Briefly, peripheral lung lobes were cut into 1 mm blocks and cultured at 37 °C and 5% CO_2_ in DMEM (HyClone; Cytiva) supplemented with 20% FBS (Gibco; Thermo Fisher Scientific, Inc.). Following 60 h of incubation, the tissues were removed and primary cells were cultured in DMEM with 10% FBS. MLVECs were passaged three or four times and used in the experiments. For a cyclic stretch, MLVECs were seeded onto collagen I-coated Bioflex^®^ 6-well culture plates. The cells transfected with MMP-9 siRNA were incubated for 48 h and the medium was changed. NF-κB signalling inhibitor BAY11-7082 (cat.no. S2913; Selleckchem, Houston, TX, USA) was administrated before cyclic stretch. The cells received cyclic stretch at 20% linear elongation for 4 h using the Flexcell^®^ FX-5000 Tension system. The control MLVECs were also cultured on BioFlex plates but were not subjected to any stretch.

### Western blotting

The protein samples were obtained from lung tissues or MLVECs using RIPA lysis buffer containing proteinase inhibitor cocktail (Beyotime Institute of Biotechnology) and the protein concentration was measured using the BCA protein assay (Beyotime Institute of Biotechnology). Equal amounts of samples (40 μg) were resolved by electrophoresis using 8% SDS polyacrylamide gels and the proteins were subsequently transferred onto PVDF membranes (0.45 μm; EMD Millipore). Immunoblotting was performed using primary antibodies against MMP-9 (cat.no. ab38898; 1:1000; Abcam), VE-cadherin (cat.no. ab205336; 1:500; Abcam), ZO-1 (cat.no. 21773-1-AP; 1:1000; ProteinTech Group, Inc.), NF-κB p65 (cat.no. 3034S; 1:1000; Cell Signalling Technology, Inc.), phosphorylated (p)-NF-κB p65 (cat.no. 3033S; 1:1000; Cell Signalling Technology, Inc.), IκB-α (cat.no. 9242S; 1:1000; Cell Signalling Technology, Inc.), and β-actin (cat.no. A1978; 1:1000; Sigma-Aldrich; Merck KGaA), followed by incubation with horseradish peroxidase-linked secondary antibodies (HRP-Goat Anti-Mouse cat.no. SA00001-1; 1:5000; HRP-Goat Anti-Rabbit; SA00001-2; 1:5000; ProteinTech Group, Inc.).

### Gelatine zymography

The enzymatic activity of MMP-9 was measured by gelatine zymography following instructions provided by the manufacturer (RTD6143, Real-Times Biotechnology Co., Ltd., Beijing, China). Briefly, proteins were mixed with loading buffer (with no reducing agent) and separated in 10% SDS polyacrylamide gelatine impregnated gels. After electrophoresis, gels were incubated with renaturation buffer for 30 min followed by incubated in incubation buffer at 4 °C overnight. The bands were visualized by Fastblue staining solution for 2 h and destained with distilled water for 1 h. The presence of enzyme activity was confirmed by the appearance of white bands on blue background.

### Statistical analysis

All data are presented as the mean ± SEM. Statistical differences between two groups were determined using a paired Student’s *t*-test (two-tailed). Statistical significance among multiple groups was determined using a one-way ANOVA followed by Bonferroni's *post-hoc* test. *p* < 0.05 was considered to indicate a statistically significant difference. The data were analyzed using SPSS 22.0 software (IBM Corp.).

## Results

### Salidroside reverses the downregulated expression levels of VE-cadherin and ZO-1 in MLVECs exposed to cyclic stretch

**Figure 1.**
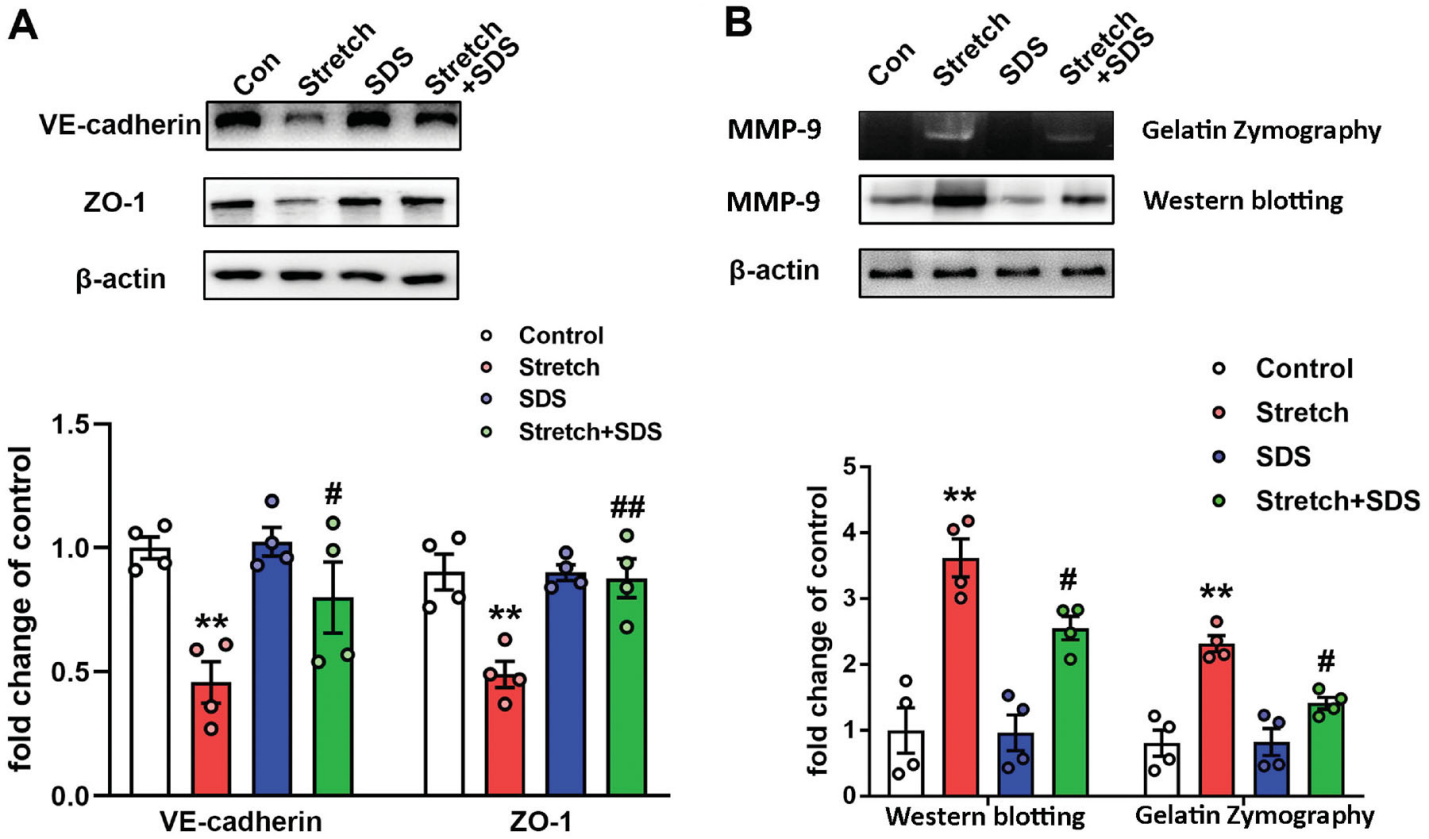
The expression of different protein levels in primary MLVECs. (A) Relative VE-cadherin and ZO-1 protein levels in primary MLVECs exposed to cyclic stretch for 4 h. Salidroside was administrated before cyclic stretch. Representative protein bands were presented on the top of the histograms. Data are presented as the mean ± SEM (*n* = 4). (B) MMP-9 activity and relative protein level treated with mechanical stretch for 4 h. Salidroside was administrated before cyclic stretch. Gelatinase activity and representative protein bands were presented on the top of the histograms. Data are presented as the mean ± SEM (*n* = 4). **p < 0.01 *vs.* control group; ^#^*p* < 0.05, ^##^*p* < 0.01 *vs.* ventilation group. MLVECs: mouse lung vascular endothelial cells; SDS: salidroside.

VE-cadherin and ZO-1 are two important proteins expressed in endothelial cells that maintain and control vascular integrity (Komarova et al. 2017). The present study explored the effects of salidroside on mechanical stretch-induced MLVECs. MLVECs were exposed to cyclic stretch, which resulted in the downregulation of VE-cadherin and ZO-1 expression. Pre-treatment of salidroside before cyclic stretch increased the levels of VE-cadherin and ZO-1 (Figure 1(A)).

### Salidroside inhibits mechanical stretch-induced upregulation of MMP-9 in MLVECs

MMP-9 has been reported to be increased in stretched cells (Cha and Purslow 2010). In the present study, the effects of stretch injury and salidroside pre-treatment on the activity and expression of MMP-9 were assessed using gelatine zymography and western blot analysis. The results indicated that pre-treatment with salidroside significantly decreased the enzymatic activity and expression levels of MMP-9 following stretch injury in MLVECs (Figure 1(B)).

### MMP-9 induces downregulation of VE-cadherin and ZO-1 expression in MLVECs exposed to cyclic stretch

**Figure 2.**
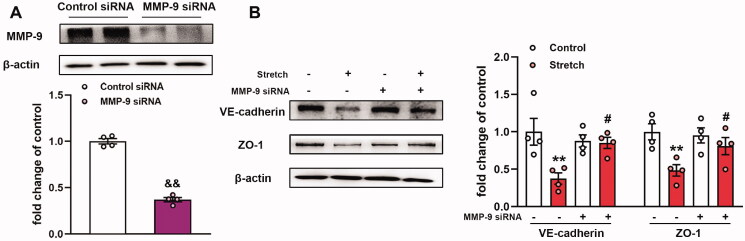
Effects of MMP-9 knockdown on the expression level of VE-cadherin and ZO-1 in primary MLVECs. (A) MMP-9 siRNA achieved a 70% decrease in MMP-9 expression at the protein level. Representative protein bands were presented on the top of the histograms. Data are presented as the mean ± SEM (*n* = 4). (B) The expression of VE-cadherin and ZO-1 protein levels in MMP-9 siRNA-treated MLVECs. Representative protein bands were shown on the left of the histograms. Data are presented as the mean ± SEM (*n* = 4). ^&&^*p* < 0.01 *vs.* control siRNA group, **p < 0.01 *vs.* control group, ^#^*p* < 0.05 *vs.* ventilation group.

MMPs can cleave the majority of the matrix proteins (Stamenkovic 2003) and non-matrix targets, such as TJ proteins, in endothelial cells (Feng et al. 2011). To explore the role of MMP-9 in stretch-induced effects on VE-cadherin and ZO-1 expression levels, MMP-9 siRNA was transfected into MLVECs for 48 h before cyclic stretch. MMP-9 siRNA achieved a 70% decrease in MMP-9 protein expression compared with the expression noted in the control siRNA group (Figure 2(A)). The results indicated that MMP-9 siRNA transfection reversed the expression levels of VE-cadherin and ZO-1 caused by mechanical stretch (Figure 2(B)). Collectively, the data indicated that MMP-9 mediated the downregulation of the expression levels of VE-cadherin and ZO-1 in MLVECs exposed to cyclic stretch.

### Salidroside induces upregulation of the expression of VE-cadherin and ZO-1 proteins in VILI

**Figure 3.**
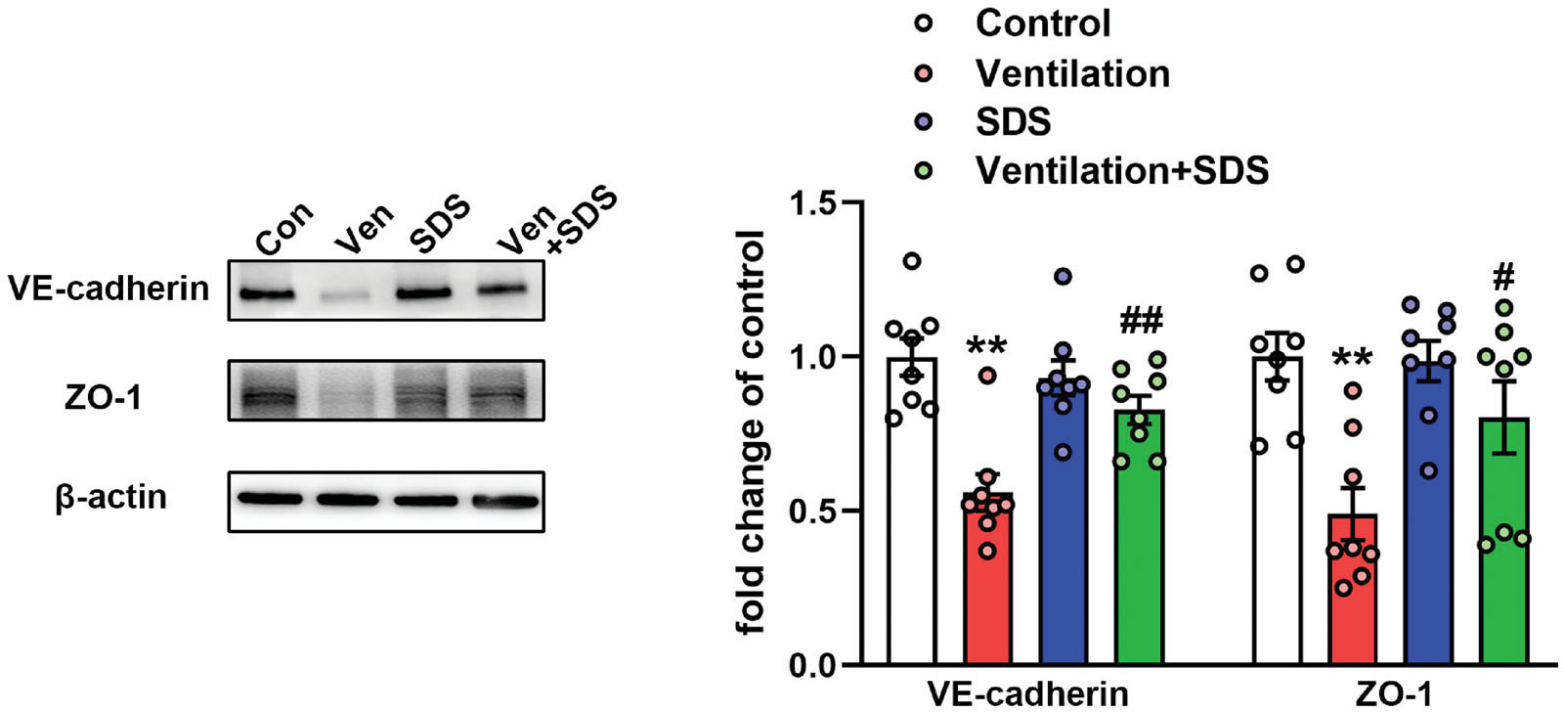
Effects of salidroside on the expression of VE-cadherin and ZO-1 in ventilator-induced lung injury model. Mice were ventilated for 4 h and salidroside (50 mg/kg) was administrated 1 h before ventilation, VE-cadherin and ZO-1 were detected by western blot analysis. Representative protein bands were presented on the left of the histograms. Data are presented as the mean ± SEM (*n* = 8). **p < 0.01 *vs.* control group, ^#^*p* < 0.05, ^##^*p* < 0.01 *vs.* ventilation group.

To explore the role of salidroside in VILI, the mice were ventilated for 4 h and salidroside (50 mg/kg) was administered 1 h before ventilation. Subsequently, the expression levels of VE-cadherin and ZO-1 were assessed in the pulmonary tissues of ventilated mice. As shown in Figure 3, MV disrupted the endothelial connection, which was indicated by the downregulation of VE-cadherin and ZO-1 protein expression levels, which were reversed by salidroside treatment.

### Salidroside inhibits the activation of the NF-κB/MMP-9 axis in ventilator-associated lung injury

**Figure 4.**
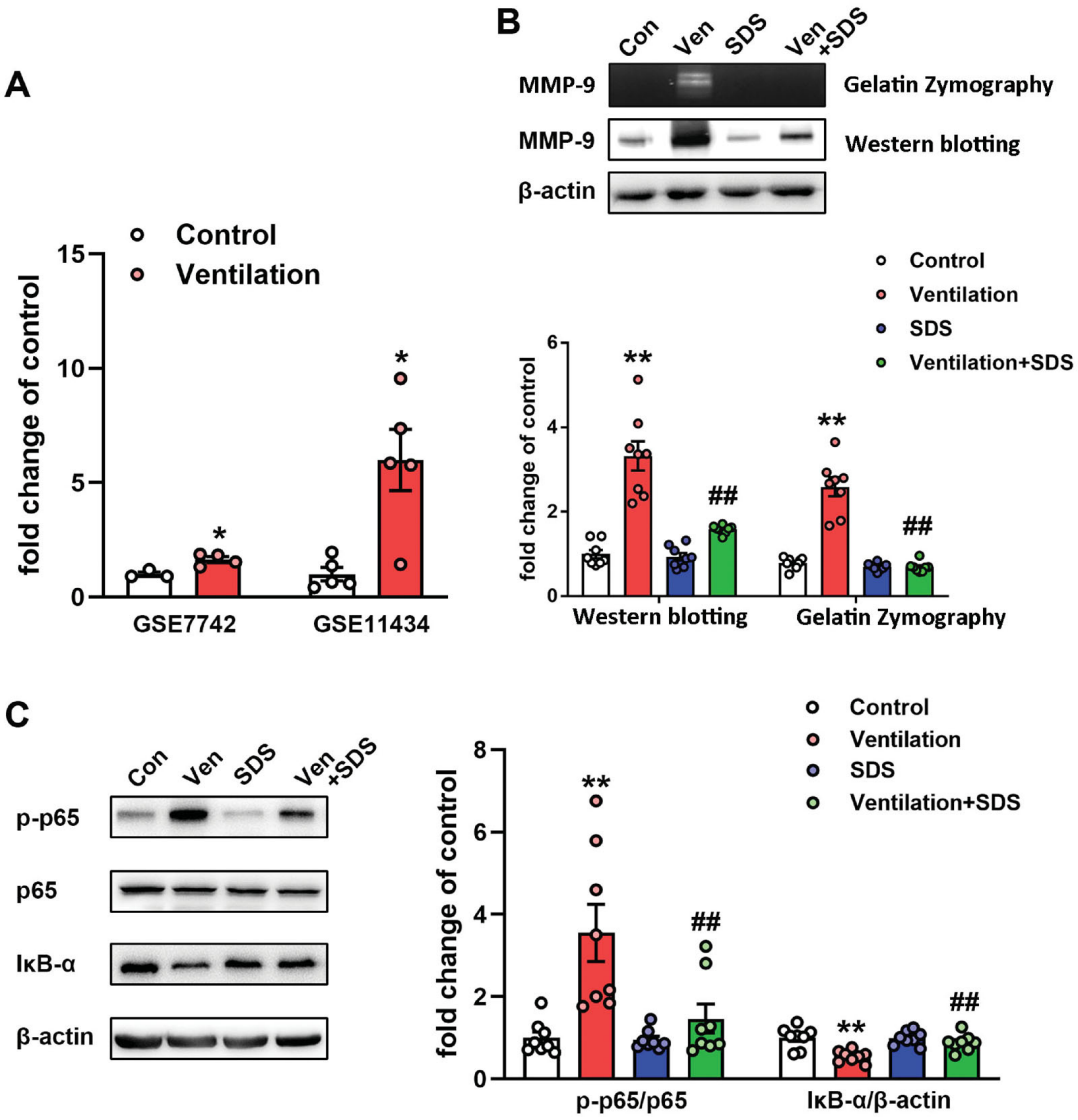
Effects of salidroside on the NF-κB/MMP-9 pathway in the model of ventilator-associated lung injury. (A) mRNA expression levels of MMP-9 in ventilated mouse lung tissues. Data were obtained from the following Gene Expression Omnibus datasets: GSE7742, GSE11434. (B) MMP-9 activity was detected by gelatine zymography, the protein level of MMP-9 was determined by western blotting. The representative bands were shown. Data are presented as the mean ± SEM. (C) The representative protein bands of the phosphorylated NF-κB p65 subunit (p-p65) and IκB-α were presented on the left of the histograms. P-p65 levels were normalized to total p65 expression. Data are presented as the mean ± SEM (*n* = 8). *p < 0.05, **p < 0.01 *vs.* control group, ^##^*p* < 0.01 *vs.* ventilation group.

**Figure 5.**
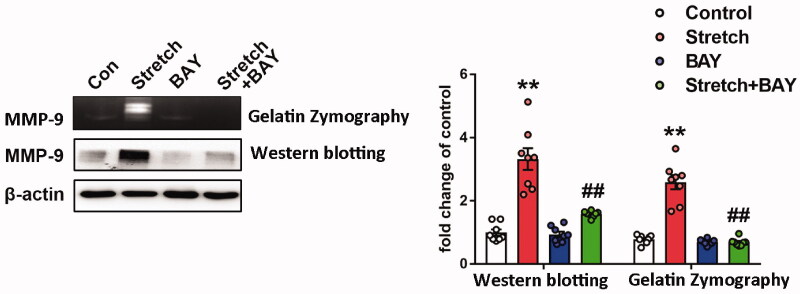
Effects of BAY11-7082 on the expression of MMP-9. MMP-9 activity and expression were analyzed by zymography (upper band) and western blotting (lower band), respectively. Indicative bands were presented on the left of histograms. Data are presented as the mean ± SEM (*n* = 4). **p < 0.01 *vs.* control group, ^##^*p* < 0.01 *vs.* ventilation group.

Increased MMP-9 expression levels were noted in pulmonary tissues of ventilated mice (Doroszko et al. 2010). Subsequently, the gene expression profiles of the GEO mouse VILI datasets GSE7742 (Dolinay et al. 2008) and GSE11434 (Wray et al. 2009) were explored. The results revealed that the mRNA expression levels of MMP-9 were significantly upregulated in mouse pulmonary tissues exposed to ventilation compared with those of the control group (Figure 4(A)). MMP-9 is involved in the pathogenesis of ALI and is regulated by NF-κB (Kang et al. 2001). In the present study, the enzymatic activity and protein expression levels of MMP-9 were significantly upregulated in the ventilated group compared with the corresponding activity and levels noted in the control group. In addition, NF-κB activation and inhibition of IκB-α activity were observed in the lung tissues of ventilated mice, whereas salidroside administration markedly inhibited the activity and expression of MMP-9 challenged by VILI (Figure 4(B)). Moreover, salidroside pre-treatment notably inhibited the phosphorylation of NF-κB and the reduction in IκB-α expression levels (Figures 4(C,D)). To further identify the association between MMP-9 and the NF-κB signalling pathway, MLVECs were treated with the NF-κB inhibitor BAY11-7082 (10 μM). Cyclic stretch-induced both the activity and expression of MMP-9, whereas administration of BAY11-7082 notably decreased activity and the expression of MMP-9 (Figure 5).

### Salidroside induces downregulation of MMP-9 expression and upregulation of VE-cadherin and ZO-1 expression in endothelial cells of pulmonary tissue

**Figure 6.**
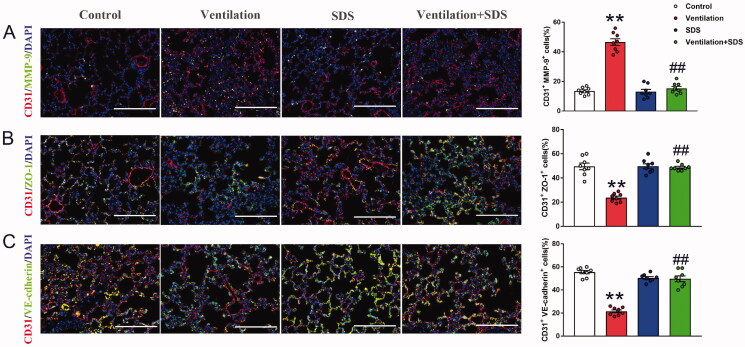
Effects of salidroside on changes of MMP-9, ZO-1, and VE-cadherin expression in endothelial cells of pulmonary tissue. (A) Lung tissues were stained with MMP-9 (green) and CD31 (red), the percent of CD31^+^MMP-9^+^ cells were shown as histograms on the right. Nuclei were counterstained with 4′,6-diamidino-2-phenylindole (DAPI) (blue). (B) Lung tissues were stained with ZO-1 (green) and CD31 (red), the percent of CD31^+^ZO-1^+^ cells were shown as histograms on the right. Nuclei were counterstained with 4′,6-diamidino-2-phenylindole (DAPI) (blue). (C) Lung tissues were stained with VE-cadherin (green) and CD31 (red), the percent of CD31^+^VE-cadherin^+^ cells were shown as histograms on the right. Nuclei were counterstained with 4′,6-diamidino-2-phenylindole (DAPI) (blue). Original magnification, ×400. Scale bar indicates 100 μm. Data are presented as the mean ± SEM (*n* = 8). **p < 0.01 *vs.* control group, ^##^*p* < 0.01 *vs.* ventilation group.

To further identify the changes of MMP-9, ZO-1, and VE-cadherin in endothelial cells of pulmonary tissue, endothelial cell marker CD31 was con-stained with MMP-9, ZO-1, and VE-cadherin, respectively and the results showed that MMP-9 expressed in endothelial cells was significantly higher in a ventilated group compared with the control group as indicated by increased percent of CD31^+^MMP9^+^ cells, pre-treatment with salidroside inhibited the positive cells of MMP-9 and CD31 (Figure 6(A)). Meanwhile, mechanical ventilation disrupted endothelial connection as indicated by decreased percent of CD31^+^ZO-1^+^ and CD31^+^VE-cadherin^+^ cells in pulmonary tissue, which were reversed by salidroside administration (Figures 6(B,C)).

### Salidroside protects against VILI

**Figure 7.**
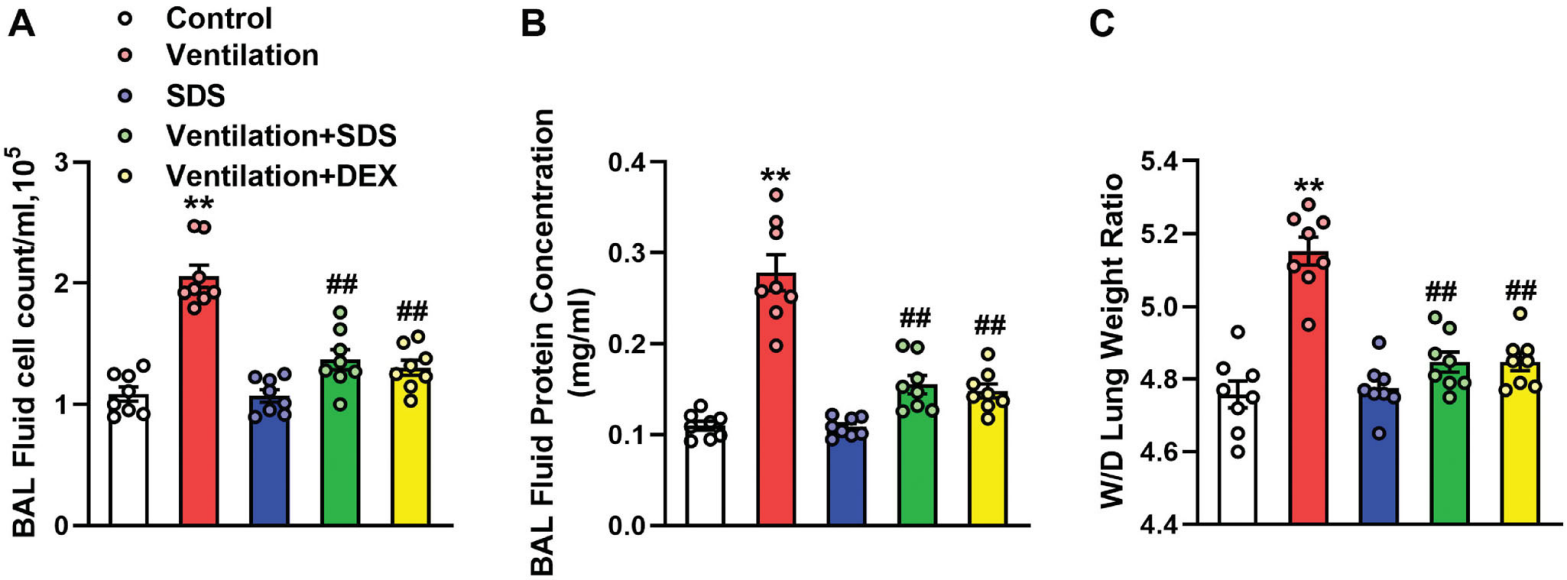
Effects of salidroside on ventilation-induced vascular hyperpermeability. (A) Cell count and (B) protein concentration in BAL fluid. (C) Lung W/D was calculated as an index of pulmonary edoema. Data are presented as the mean ± SEM (*n* = 8), **p < 0.01 *vs.* control group, ^##^*p* < 0.01 *vs.* ventilation group. BAL: bronchoalveolar lavage; W/D: wet-to-dry weight ratio.

**Figure 8.**
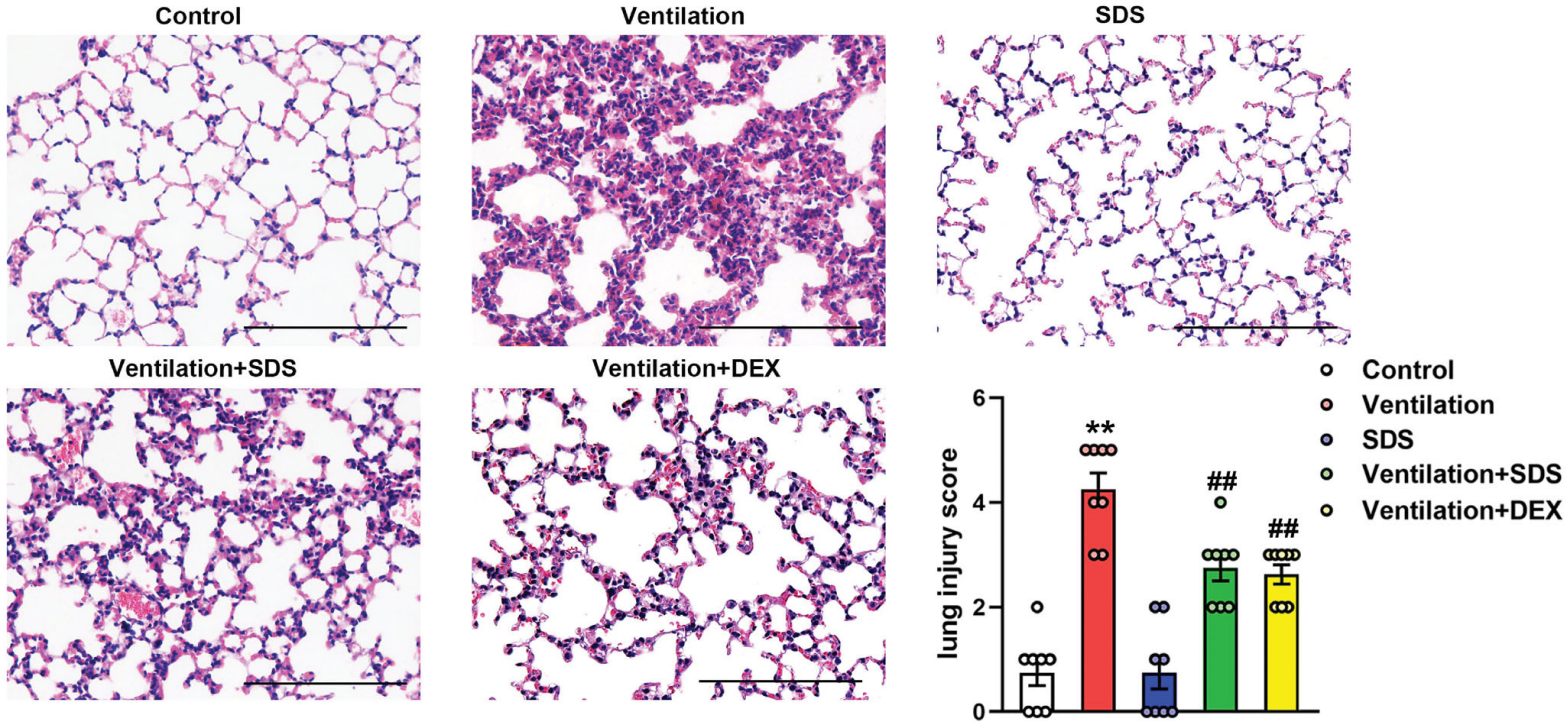
Effects of salidroside on ventilation-induced lung injury. Representative haematoxylin and eosin staining of lung tissues. The severity of lung injury was scored to quantify the degree of lung pathology. Original magnification, ×200. Scale bar, 100 µm. Data are presented as the mean ± SEM (*n* = 8). **p < 0.01 *vs.* control group, ^##^*p* < 0.01 *vs.* ventilation group.

As shown in Figures 7(A,B), MV resulted in significant impairment in endothelial permeability, which was illustrated by significant increases in cell counts and protein expression levels in BALF. These effects were inhibited by the treatment of salidroside and Dex. In addition, ventilation-induced a higher W/D weight ratio compared with that of the control group, which was markedly improved in salidroside and Dex-treated mice (Figure 7(C)). Subsequently, the effects of salidroside on the pathological changes induced by VILI were investigated using haematoxylin and eosin (H&E) staining. As shown in Figures 8(A,B), MV exhibited more severe edoema, thickening of the alveolar wall, and neutrophil infiltration in the lung parenchyma, whereas these effects were markedly reduced by administration of salidroside and Dex.

## Discussion

VILI is characterized by pulmonary inflammation and alveolar membrane hyperpermeability (Zhang et al. 2014). The primary causes of the hyperpermeability noted in the alveolar membrane are the destruction of the alveolar membrane and the decreased expression of junction proteins. Interendothelial junctional complexes are composed of AJs and TJs that maintain and control the integrity of the endothelial barrier (Bazzoni and Dejana 2004). VE-cadherin is a critical determinant of adherens integrity, which is a special cadherin protein that appears in all endothelial cells of the vessels. ZO-1 is the primary TJ protein linking TJs to the actin cytoskeleton (Komarova et al. 2017). Mechanical stretch induces the downregulation of intercellular junction protein expression, resulting in basement destruction and further tissue damage (Tian et al. 2016). MMPs have been demonstrated to hydrolyze proteins of AJs and TJs, resulting in endothelial dysfunction (Lindsey et al. 2006). Among them, MMP-9 has gained considerable attention about the progression of ALI owing to its conflicting data in the development of ALI. The lack of MMP-9 expression reduced VILI by decreasing alveolar neutrophilic infiltration, possibly by modulation of the cytokine response in the air space (Albaiceta et al. 2008). MMP-9 knockout mice developed exacerbated lung injury during infection (Renckens et al. 2006) and enhanced allergen-induced airway inflammation (McMillan et al. 2004). It has been shown that inhibition of MMP-9 improves survival in a sepsis-associated lung injury model (Steinberg et al. 2003) and protects against the development of VILI through downregulation of neutrophil-mediated inflammation (Fligiel et al. 2006; Kim et al. 2006).

MMP-9 belongs to a family of zinc-dependent endopeptidases that can cleave a variety of substrates (Beroun et al. 2019). It has been previously shown that MMP-9 cleaves IL-1β (Ito et al. 1996) and platelet-derived CD40L, which is known to regulate neutrophil recruitment and lung damage (Rahman et al. 2013). Previous evidence has shown that MMP-9 activation leads to degradation of ZO-1 and VE-cadherin and consequent disruption of the blood-brain barrier (BBB) integrity (Abdul Muneer et al. 2012; Wu et al. 2019). Feng et al demonstrated that the upregulation of MMP-9 expression in mouse brain tissues was accompanied by the downregulation of ZO-1 expression when the permeability of the BBB was increased (Feng et al. 2011). Similarly, it was shown that mechanical stretch decreased the expression of VE-cadherin and ZO-1, whereas it upregulated the expression levels of MMP-9. Furthermore, inhibition of MMP-9 attenuated vascular hyperpermeability and prevented gap formation and TJ rearrangement in cerebral endothelial cells (Bauer et al. 2010). It was hypothesized that downregulation of the expression levels of VE-cadherin and ZO-1, which was induced from the mechanical stretch, may be attributed to MMP-9 activation. As expected, knockdown of MMP-9 in primary cultured MLVECs resulted in increased expression of VE-cadherin and ZO-1 following exposure to cyclic stretch. MMP-9 mediates the degradation of VE-cadherin and ZO-1 in mechanical stretch-induced endothelial dysfunction.

Salidroside exerts a protective effect in numerous lung injury models. For example, salidroside protects against bleomycin-induced pulmonary fibrosis and has been shown to inhibit IκBα phosphorylation and NF-κB p65 nuclear accumulation (Tang et al. 2016). In addition, salidroside ameliorated the histopathological changes in the lung of CLP-ALI rats and reduced CLP-induced lung permeability. These effects were also accompanied by increased peroxisome proliferator-activated receptor-γ levels and IκB activation and decreased NF-κB p65 activation in lung tissues (Liu et al. 2014). A previous study demonstrated that salidroside may confer protection against ventilation-induced lung injury *via* sirtuin 1-dependent inhibition of NLR family pyrin domain containing 3 inflammasome (NLRP3) activation (Wang et al. 2017). In the present study, pre-treatment of salidroside inhibited the expression of MMP-9, former studies demonstrated that targeting the NLRP3 inflammasome with inhibitor MCC950 prevents MMP-9 activation in macrophages (Ren et al. 2020) and human umbilical vein endothelial cells (Chen et al. 2017), whether NLRP3 mediates upregulation of MMP-9 in stretched MLVECs needs further validation. Salidroside also reversed VE-cadherin and ZO-1 levels both in stretched MLVECs and ventilated pulmonary tissues. Considering that MMP-9 mediated the downregulation of VE-cadherin and ZO-1 in stretched MLVECs, it was hypothesized that salidroside may exert protective effects in ventilation-induced endothelial dysfunction *via* the inhibition of MMP-9. It has been reported that the NF-κB-binding site, located at −600 bp in the human MMP-9 promoter, renders the MMP-9 gene responsive to TNF-α (Mercurio and Manning 1999) and blocks NF-κB nuclear translocation, which in turn reduces the expression of MMP-9 in human fibrosarcoma cells (Yan et al. 2001). It was shown that salidroside inhibited NF-κB phosphorylation and IκBα reduction in ventilation-induced lung tissues, which inhibited the NF-κB signalling pathway. BAY11-7082 markedly reduced the expression of MMP-9, whereas salidroside could suppress the expression of MMP-9 in VILI by inhibiting NF-κB p65 activation. Additional studies are required to confirm these findings.

## Conclusions

In the present study, the protective effects of salidroside were assessed in ventilation-induced lung injury and vascular endothelial disruption. This effect was possibly mediated by inhibition of the expression of MMP-9. The data provided novel insights into the role of MMP-9 in VILI and described a novel mechanism of ventilator-induced disruption of endothelial junction proteins. In this respect, it is expected that salidroside can be used to protect against stretch-induced endothelial barrier function as well as ventilation-induced lung injury.
